# Clinical analysis of reversible splenial lesion syndrome in Chinese adults: A retrospective study of 11 cases

**DOI:** 10.1097/MD.0000000000022052

**Published:** 2020-09-04

**Authors:** Xiaoyu Gao, Qiaochan Feng, Saeed Arif, Jahanzeb Liaqat, Bing Li, Kun Jiang

**Affiliations:** a Department of Neurology, the Affiliated Yantai Yuhuangding Hospital of Qingdao University, Yantai, China; b Neurology Department, Pakistan Emirates Military Hospital, Rawalpindi, Pakistan.

**Keywords:** adult, corpus callosum, magnetic resonance imaging, reversible lesion, splenium

## Abstract

Reversible splenial lesion syndrome (RESLES) is a clinico-radiological entity that defines a reversible lesion in the splenium of the corpus callosum (SCC) on magnetic resonance imaging (MRI). The clinical and radiological characteristics of RESLES are poorly defined and most RESLES literature is in the form of case reports. We reviewed the clinical and radiological data from 11 RESLES patients in order to more clearly describe the characteristics of this disorder in adults.

Patients included in this study were diagnosed with RESLES from May 2012 to March 2018. We collected clinical, imaging, and laboratory data of 11 adult patients from Neurology Department of the Affliated Yantai Yuhuangding Hospital of Qingdao University. After analyzing various clinico-radiological features and laboratory parameters, including serum sodium, pathogen testing, cerebrospinal fluid (CSF) studies, electroencephalography (EEG), and MRI findings, we made a diagnosis of RESLES based on the criteria proposed previously by Garcia-Monco et al.

Of the 11 patients, 7 (63.63%) were male and 4 (36.36%) were female, ranging in age from 24 to 62 years with an average age of 31.48 ± 11.47 years. Seven cases occurred in the months of winter and spring (December–March). The primary clinical symptoms were headache, seizure, disturbance of consciousness, mental abnormality, and dizziness. All 11 patients had lesions in the SCC and all the lesions disappeared or significantly improved on follow-up imaging that was done within a month of symptom resolution.

We found 5 (45.45%) patients had a CSF opening pressure >180 mmH_2_O, in addition to elevated protein and(or) leukocytes levels in 3 (27.27%) patients. The serum sodium concentration in 6 (54.55%) patients was low (<137 mmol/L) and EEG showed nonspecific slowing in waves 4 (36.36%) patients.

When we encounter clinical manifestations such as headache accompanied with mental symptoms, disturbance of consciousness or epilepsy, and brain MRI finds lesions of the corpus callosum, we should consider whether it is RESLES. In order to find out the possible cause of the disease, we should carefully inquire about the history of the disease, complete etiology examination, and CSF tests. Of course, it is one of the necessary conditions for the diagnosis that the lesions in the corpus callosum are obviously relieved or disappeared.

## 1 Introduction

The magnetic resonance imaging (MRI) finding of a reversible lesion in the central portion of the splenium of the corpus callosum (SCC) without any accompanying lesions has been reported in patients with epilepsy-receiving antiepileptic drugs. ^[1]^ In 2004, the concept of “Mild encephalitis/encephalopathy with reversible splenial lesion” (MERS) was proposed. ^[2]^ In 2011, Garcia-Monco et al^[3]^ furthered this concept by proposing the reversible splenial lesion syndrome (RESLES) based on previous studies, which included reversible corpus callosum lesions of varying etiologies.

The clinical course of RESLES is typically benign, except in patients with a severe, underlying disorder. The characteristic lesion of RESLES occurs in the SCC, which is best evaluated using brain MRI. Importantly, these lesions disappear or improve significantly upon follow-up imaging.

RESLES is a clinical-imaging syndrome. The clinical symptoms of this disease lack specificity and most patients have a good prognosis. RESLES has been previously reported in scientific literature, but there have been no large-scale clinical studies in adults. The precise frequency of RESLES is unknown, and mechanisms explaining the predilection for the SCC are yet to be proposed. We retrospectively reviewed 11 cases of adults with RESLES and analyzed their clinical, imaging, and laboratory characteristics to better understand this disease.

## 2 Methods

### 2.1 Patient Characteristics

We obtained the clinical, imaging, and laboratory data of 11 adult patients from Neurology Department of the Affliated Yantai Yuhuangding Hospital of Qingdao University, who were diagnosed with RESLES from May 2012 to March 2018. Diagnosis was based on the criteria proposed by the Garcia-Monco et al,^[3]^ which included patients presenting with neurological deficits and brain MRI lesions in the SCC with or without extra-callosal lesions, which either disappeared or significantly improved during follow-up studies. The presence of additional brain lesions or other involved regions of the corpus callosum (CC) were considered, as long as the primary lesion was located on the SCC.

Patients with persistent splenial lesions or those with missing follow-up data were excluded from this study. Patients with acute disseminated encephalopathy (ADEM) or other common demyelinating disorders involving the CC were also excluded.^[3]^

All patients underwent emergency biochemical examination on the day of consultation. Within 2 days of consultation, 8 of 11 patients also (including 7 patients with fever) underwent serological antibody testing for *Toxoplasma gondii* virus IgG and IgM, Cytomegalovirus IgG and IgM, Rubella virus IgG and IgM, Herpes Simplex virus IgG and IgM, Epstein--Barr virus IgG and IgM, Legionella Pneumophila IgM, *Mycoplasma pneumoniae* IgG and IgM, Rickettsia Q fever IgM, *Chlamydia pneumoniae* IgM, Adenovirus IgM, and Respiratory Syncytial virus IgM. Throat swabs were used for detection of Influenza A.

Eight of 11 patients received a lumbar puncture within 2 days of hospitalization. Of the remaining 3, 1 patient did not consent procedure and 2 did not undergo the procedure, which included a history of syncope and glossopharyngeal neuralgia. Tests conducted on CSF samples included bacterial culture, Gram staining, India Ink staining, and modified acid-fast staining. The CSF samples were also tested for herpes simplex viruses 1 and 2 using polymerase chain reaction (PCR).

All 11 patients received EEG and brain MRI, but only 8 patients underwent contrast enhanced brain imaging. All patients also received follow-up brain MRI after clinical symptoms improved or disappeared.

### 2.2 Clinical symptoms and image evaluation

The patients’ prognoses, clinical symptoms, and laboratory examinations were analyzed by 2 senior neurologists. The brain MRI images were analyzed by 2 senior neuroimaging diagnosticians/radiologists.

### 2.3 Statistical analysis

The patient data were analyzed using SPSS20.0 statistical analysis software and the quantitative data are expressed as mean ± standard deviation (Table 1).

**Table 1:**
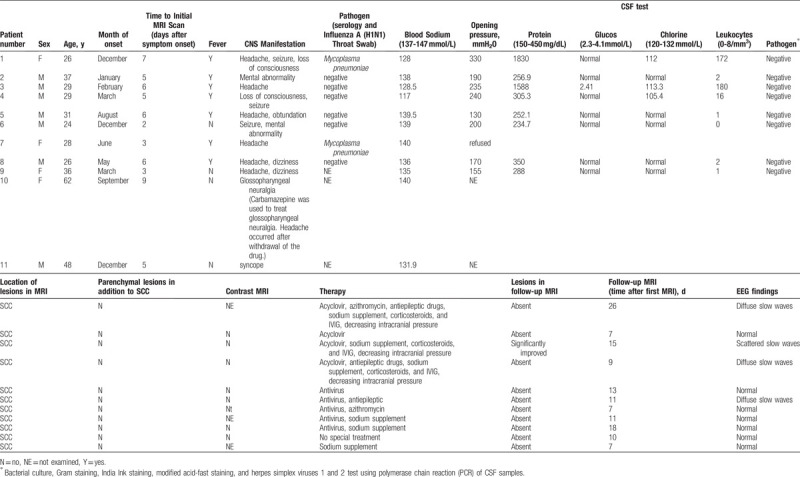
Clinical features, laboratory tests, EEG, CSF and MRI findings, therapy and prognoses of all 11 patients.

## 3 Results

### 3.1 Clinical features

Eleven patients (7 male and 4 female) were included in this study. They ranged in age from 24 to 62 years, with an average age of 31.48 ± 11.47. Seven cases occurred in the winter and spring months.

Fever was the primary clinical symptom documented in 7 patients, followed by headache, which was reported by six patients. Seizures were present in 3 patients and 4 patients experienced either loss of consciousness or mental abnormality. Other clinical symptoms included dizziness (2 patients), obtundation (1 patient), and syncope (1 patient). All patients’ clinical symptoms showed marked improvement or disappeared entirely within 1 month of hospitalization.

### 3.2 CSF findings

Eight of 11 patients received a lumbar puncture. An opening pressure of >180 mmH_2_O was documented in only 5 (45.45%) patients. Leukocytes were elevated in 3 (27.27%) patients, and proteins were elevated in 2 patients (18.18%). CSF chlorine levels were reduced in 3 (27.27%) patients.

### 3.3 Laboratory tests

All patients underwent emergency biochemical examinations on the day of consultation. Initial serum sodium concentration in 6 (54.55%) patients was low (< 137 mmol/L) but normalized after treatment. Two cases were positive for *M. pneumonia* antibody.

### 3.4 Electroencephalography (EEG)

**Figure 1:**
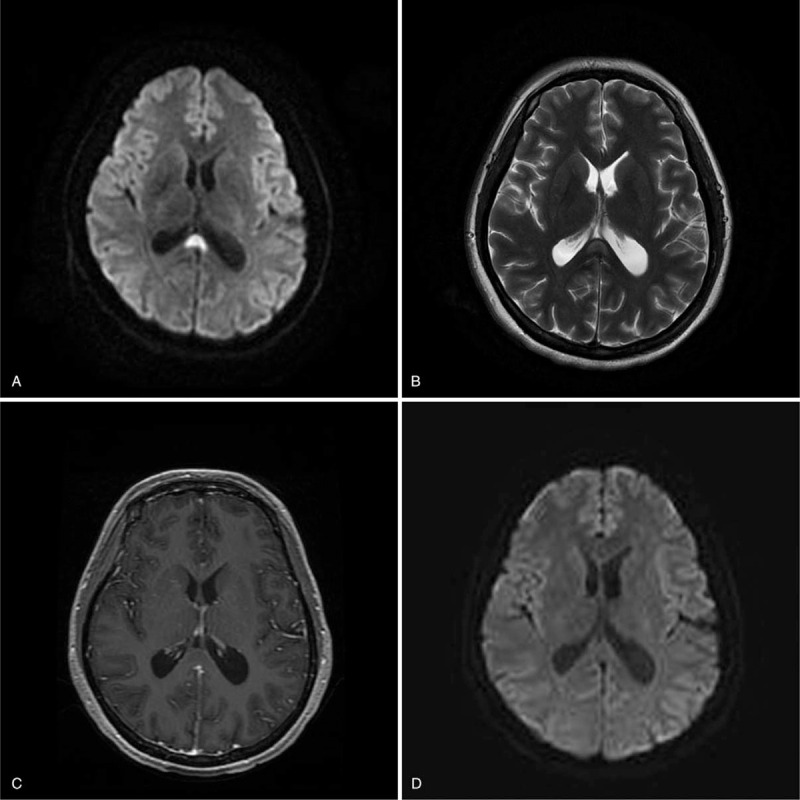
In case 1, a 26-year-old woman presented with fever and headache. The initial enhanced magnetic resonance 3.0 T MRI indicated a region of the splenium hyperintensities on the DWI image (A) and FLAIR (B), without contrast enhancement effect (C). The follow-up 1.5 T MRI at 26 days later indicated the complete disappearance of the splenial lesion (D).

**Figure 2:**
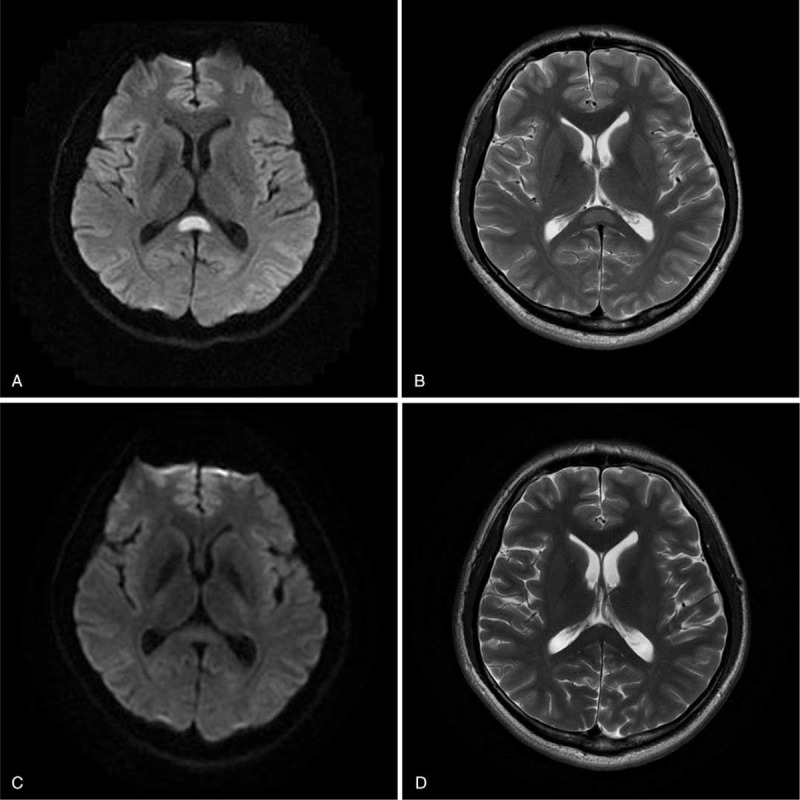
In case 3, a 29-year-old man presented with fever and headache. This patient was clinically diagnosed with tuberculous meningitis. The initial 3.0 T MRI indicated a lesion in the splenium and a hyperintensity on the DWI (A) and T2-weighted (B) images; The follow-up1.5 T MRI indicated the resolution of the splenial lesion at 15 days after the initial MRI examination (C,D).

All 11 patients underwent electroencephalography (EEG) examination. Diffuse slow waves were detected in 3 (27.27%) patients, while 1 (9.09%) patient had scattered slow waves. The EEG of the other 7 (63.63%) patients was normal (Figs. 1 and 2).

### 3.5 MRI findings

The average time from onset of symptoms to initial brain MRI was 5.18 ± 1.99 days. The primary MRI abnormalities were high signal intensity in the CC on T2-weighted images (T2WI), fluid-attenuated inversion recovery images (FLAIR), diffusion-weighted images (DWI), and low or equal signal intensity on T1-weighted imaging (T1WI), and no gadolinium enhancement in SCC on T1WI postcontrast sequences. The lesions in all 11 patients were localized to only the SCC (see Figs. 1 and 2).

All patients received a follow-up brain MRI when clinical symptoms improved significantly or disappeared entirely. This imaging occurred on average 12.18 ± 5.76 days after initial imaging. The initially detected abnormal signal was absent in 10 cases and lesion range/size decreased in the remaining patient, suggesting the reversibility of the lesions.

### 3.6 Treatment and Prognosis

Ten patients received treatment according to either the presumed etiology of their condition or laboratory test results, which included acyclovir, azithromycin, anti-epileptic drugs (AEDs), and correction of electrolyte imbalance (serum sodium). Three patients were treated with corticosteroids and IVIG because of their critical condition. No specific treatment was given to the remaining patient. The clinical symptoms and MRI of the 11 RESLES patients showed resolution within 1 month.

## 4 Discussion

We received the cases of 7 (63.63%) males and 4 (36.36%) females who experienced RESLES. The average age of the patients was 34.18 ± 11.47 years. Subgroup analysis revealed that 9 (81.81%) cases occurred in young adults aged between 24 and 37 years, while the remaining 2 patients were middle-aged/elderly (ages 48 and 62 years). This finding is not entirely unusual however, as most RESLES cases reported previously occurred in children.

Garcia-Monco et al^[3]^ previously summarized the most common causes of RESLES. These include infection, epilepsy or withdraw of antiepileptic drugs, nutritional and metabolic factors, Charcot Marie-Tooth disease, high-altitude cerebral edema, systemic lupus erythematosus, and others.^[3]^ Anti-VGKC (human voltage-gated potassium channel) autoantibodies have been reported in RESLES patients.^[4]^ Other studies have implicated the role of genetic factors in the development of RESLES.^[5]^

In this study, 7 of 9 young patients had fever, and all 7 underwent infectious screening. However, an increase of leukocytes or leukocytes and proteins in CSF was found in only 3 cases, suggesting inflammatory changes in the CSF. Two young patients with fever had *M. pneumonia* antibodies. We think the incidence of RESLES in these young people is closely related to infection. But the cause of other young patients is not clear.

In the literature, cases of RESLES associated infection have been most often associated with viral causes. Specifically, influenza virus and rotavirus was strongly associated with mild encephalitis/encephalopathy with a reversible splenial lesion in a prior study.^[6]^ The extent of our viral testing panel was utilized in our study yet many pathogens have been associated with RESLES, including measles, herpes virus 6, Epstein--Barr virus, varicella-zoster virus, adenovirus, Salmonella enteritidis, *Escherichia coli*, *Legionella pneumophila*,^[3]^ dengue virus,^[7]^ and *M. pneumoniae*.^[8]^ A candidate mechanism for RESLES in patients with an underlying infection is related to the influx of inflammatory cells and macromolecules, combined with cytotoxic edema, which may cause reversible lesions in the SCC.^[2]^ Other studies have suggested that oxidative stress may also be involved.^[9]^

The 48- and 68-year-old patients did not present with fever, and we think the etiology of their RESLES may be different. One of these patients was treated with carbamazepine for glossopharyngeal neuralgia. After a poor therapeutic effect, carbamazepine was replaced with pregabalin and subsequent brain MRI detected lesions in the SCC. The withdrawal of carbamazepine is the most likely cause of RESLES in this case. Prior studies have shown that AED withdrawal in RESLES is more significant than the seizures themselves, ^[3]^ as many AEDs target cation channels, which can influence water balance. Sudden withdrawal of AEDs may cause transient cerebral edema because of disturbed water balance, leading to a reversible lesion in the SCC.^[10]^ The other patient in this group experienced concurrent syncope and hyponatremia. The syncope resolved after the correction of hyponatremia. Because the syncope was likely related to the patient's hyponatremia and hypovolemia, we presume hyponatremia may have caused this patient's SCC lesion.

Clinical manifestations of RELES are diverse.^[11,12]^ Clinical features of these eleven patients included headache, seizure, loss of consciousness, mental abnormality, dizziness, obtundation and syncope. In our group of adults, headache was the predominant clinical finding yet prior studies of RESLES in children showed seizure, behavior changes, and altered consciousness were the most common neurological symptoms.^[11]^ We suspect that this is related to differences in the incompletely mature central nervous system and the blood–brain barrier in children versus adults. The SCC is located at the posterior end of the CC with fibers that follow a posterior course, contribute to the forceps major, and connect the occipital lobes. Prior studies have shown that SCC lesions can present with confusion, ataxia, dysarthria, and seizure among other clinical manifestations.^[13]^ However, lesions of the SCC cannot completely explain the encephalopathy symptoms of disturbance of consciousness and mental abnormality. Previous studies classified MERS into 2 types according to imaging findings: type 1 and type 2.^[14]^ Type 1 MERS only has a solitary lesion of SCC, whereas type 2 MERS has extensive white matter and/or entire callosal lesions.^[14,15]^ A prior study reported that patients with mild encephalitis/encephalopathy with reversible splenial lesion in whom initial brain MRI revealed type 2 MERS lesions, had only an isolated SCC lesion the following day.^[14]^ Type 1 and type 2 lesions may result from MRI observations visible at different stages in the course of this disease. Therefore, we speculate that it is possible for some patients’ lesions to involve other parts of the brain outside of the SCC, resulting in a series of neurological manifestations. The time between the initial MRI and the symptom onset was not uniform in our study, ranging from 2 to 9 days, and we observed no type 2 manifestations. Thus, the proposed mechanism cannot explain all cases. Encephalopathy symptoms were also due to infection and hyponatremia.

Epilepsy is one of the most common clinical symptoms of SCC lesions.^[13]^ In this study, 3 (27.27%) patients had seizures as their chief complaint. Prior literature suggests that CC changes cannot act as a trigger for epilepsies.^[16]^ Fever, abnormal CSF and EEG findings were found in these 3 epileptic patients. We believe that encephalitis or encephalopathy caused the seizures and lead to the splenial lesions because of the brief, reversible failure of cellular fluid regulation that occur during convulsions.^[3]^ Other investigators have suggested that the prevalence of SCC abnormalities results from the robust spread of seizure activity through the splenium to bilateral hemisphere, from independent seizure foci in patients with epilepsy.^[17]^

Lesions found in RESLES are often associated with hyponatremia. A plausible mechanism is that hypotonic hyponatremia leads to the entry of water into the brain, causing brain edema. However, it is not clear why the lesions primarily involve the SCC. Earlier work has found significant differences between the sodium levels of patients with mild encephalitis/encephalopathy with reversible splenial lesion and those with of groups (e.g., upper respiratory infection, other types of encephalopathy and febrile seizures).^[18]^ We observed hyponatremia in 6 (54.55%) of our patients, a significantly lower rate than that of previous RESLES studies in children.^[19]^ Children under 16 years of age are at an increased risk for developing hyponatremic encephalopathy because of their relatively large brain to intracranial volume ratio.^[20]^ MRI examination is not routinely performed in adults with hyponatremia, because adults are less likely to have the same nervous system clinical symptoms than children with the same serum sodium levels. The difference in our incidence of patients with RESLES and hyponatremia versus prior work is likely the result of different etiological profiles of RESLES.

Leukocyte elevation in CSF can also be observed in patients with RESLES, but it is not considered specific.^[10,21]^ Of the 8 patients who underwent CSF examination, only 5 (45.45%) had abnormal findings. In the 3 (27.27%) patients, an increased leukocyte count or leukocyte count and protein accompanied the increase of brain pressure. In these 3 cases, a decrease of blood sodium levels also accompanied the increase of brain pressure. Brain edema caused by low sodium is one reason for the increase of brain pressure. Intracranial infection was considered in 2 cases, though only a mild increase of cerebral pressure was observed, and there were no abnormalities in the CSF leukocyte levels and biochemical/bacteriological examinations. CSF chlorine levels were reduced in 3 patients and were associated with hyponatremia.

Of the 11 patients who underwent EEG examinations, only 3 (27.27%) patients had diffuse slowing and 1 (9%) patient had scattered slow waves. Prior research suggests that patients with diffuse slow waves on EEG have poor outcomes, whereas the patients with occipital slow waves did not exhibit similar tendencies.^[12]^ However, our study noted no difference in the prognosis of the 3 patients with slow waves on EEG versus other patients.

Brain MRI is the optimal imaging modality for identifying lesions in the SCC. All 11 cases in our study had a singular lesion in the midline of the SCC, which resolved or significantly improved on follow-up imaging. This suggests the reversibility of these lesions and is in accordance with prior reports.^[2,3]^ Brain MRI features are usually in the form of high-signal-intensity on T2WI, FLAIR, DWI, and low or equal signal intensity on T1WI sequences. None of the 8 patients who received contrast enhanced brain MRI showed contrast enhancement in the SCC. Prior studies have suggested that decreased apparent diffusion coefficient (ADC) values on ADC maps and low ADC indicates cytotoxic edema in the SCC,^[3]^ yet several cases of vasogenic edema (high ADC values) have been reported previously.^[22–25]^ However, we could not perform ADC examinations on our patients due to technical limitations.

Despite the varying etiologies of RESLES and the proposed pathological mechanisms, it is unknown why RESLES only affects the SCC. We suggest the following reasons: The SCC is vascularized by the terminal and choroidal branches of the posterior cerebral artery, lacking sympathetic innervation (vertebrobasilar system), and the ability of cerebrovascular self-regulation compared with the internal carotid artery system. However, this cannot explain why the same lesions did not occur in other parts of the brain vascularized by the vertebrobasilar artery system. Intra-myelinic edema may result from the separation of myelin layers.^[2]^ This theory is contradicted by a report of a reversible SCC lesion in a neonate whose SCC is still completely unmyelinated.^[26]^ In this case, interstitial edema may occur, as the axons in SCC are so tightly packed that any water between the unmyelinated axons would be pathogenic.^[10]^ Because RESLES has many infectious etiologies, it may be possible that viral antigens or receptors on the antibodies induced by the antigens have specific affinities for splenial axons or the myelin sheaths surrounding them,^[2]^ but this is yet to be confirmed experimentally.

An evidence-based therapeutic regimen for RESLES has not yet been developed, because of an insufficient number of patients. As done in prior studies,^[1,27]^ 10 of our patients received treatment according to their presumed etiology and laboratory tests, including acyclovir, azithromycin, AEDs, and the correction of an electrolyte imbalance. In addition, 3 patients were treated with corticosteroids and IVIG because of their critical symptoms or CSF test results. For RESLES caused by the withdrawal of antiepileptic drugs, it appears no specific treatment is necessary. All 11 of our patients’ symptoms showed marked improvement or disappeared within 1 month, with a good prognosis and no lasting neurological sequelae.

Because of the small sample size of our study, we acknowledge that the clinical characteristics of our adult-onset RESLES may not accurately reflect that of all patients with RESLES. A more intensive study with a large sample size and prospective study design would be required to more definitively explain why this group of patients have lesions that selectively involve the CC.

## 5 Conclusion

We found that younger adults have the highest incidence of adult-onset RESLES. Nonspecific clinical manifestations such as headache were common in our study, which was often accompanied by hyponatremia. Lumbar puncture revealed the potential for CSF abnormalities such as high opening pressure, elevated leukocyte count, and elevated protein. EEG may show nonspecific slow wave abnormalities. Reversible lesions of the SCC on brain MRI remain the characteristic feature of this condition. Etiological symptomatic treatment is indicated for RESLES treatment and for severe clinical manifestations or severely abnormal CSF findings, corticosteroids and IVIG should be administered early in the course of treatment.

## 6 Availability of data and materials

Data generated during this study are included in this published article.

## Author contributions

XYG and QCF provided technical and material support, helped design the study, drafted the manuscript, participated in the patient's medical treatment, and analyzed data. SA and JL revised the paper. BL and KJ helped design the study, drafted the manuscript and provided technical and material support.

**Data curation:** xiaoyu gao.

**Investigation:** xiaoyu gao.

**Methodology:** xiaoyu gao, Qiaochan Feng.

**Resources:** xiaoyu gao.

**Supervision:** xiaoyu gao.

**Writing – review & editing:** xiaoyu gao, Qiaochan Feng.

**Writing – original draft:** Qiaochan Feng.
